# Total Body Sodium Balance in Chronic Kidney Disease

**DOI:** 10.1155/2021/7562357

**Published:** 2021-09-22

**Authors:** Kylie Martin, Sven-Jean Tan, Nigel D. Toussaint

**Affiliations:** ^1^Department of Nephrology, The Royal Melbourne Hospital, 300 Grattan Street, Parkville, Victoria 3050, Australia; ^2^Department of Medicine (RMH), University of Melbourne, Parkville, Victoria 3050, Australia

## Abstract

Excess sodium intake is a leading but modifiable risk factor for mortality, with implications on hypertension, inflammation, cardiovascular disease, and chronic kidney disease (CKD). This review will focus mainly on the limitations of current measurement methods of sodium balance particularly in patients with CKD who have complex sodium physiology. The suboptimal accuracy of sodium intake and excretion measurement is seemingly more marked with the evolving understanding of tissue (skin and muscle) sodium. Tissue sodium represents an extrarenal influence on sodium homeostasis with demonstrated clinical associations of hypertension and inflammation. Measurement of tissue sodium has been largely unexplored in patients with CKD. Development and adoption of more comprehensive and dynamic assessment of body sodium balance is needed to better understand sodium physiology in the human body and explore therapeutic strategies to improve the clinical outcomes in the CKD population.

## 1. Introduction

Excess sodium is associated with increased mortality in patients with chronic kidney disease (CKD). As sodium excretion progressively decreases with decline in kidney function, individuals with CKD are particularly sensitive to excess sodium in tissues. This review explores current challenges in accurately evaluating sodium intake and excretion in the CKD population. We advocate for a more comprehensive and dynamic assessment of total body sodium balance, particularly with the shift in body sodium paradigm to include an appreciation of tissue (skin and muscle) sodium [1]. Innovative methods are needed to measure tissue sodium in addition to the extracellular component of osmotically active sodium that is conventionally reflected in sodium intake and excretion measurements.

## 2. Main Text

### 2.1. Evolution of Sodium's Place at the Table

Sodium is an essential nutrient for humans, but the human body only requires small amounts [2]. Humans have evolved from an environment with little additive sodium and adapted to retain salt naturally occurring in food and water [3]. Salt has been used in food preservation and become an increasing addition to food with the advent of agriculture; but the human body has yet to adapt to excreting larger quantities of sodium [3]. High sodium intake is the leading dietary risk factor for mortality in the general population [4]. Most countries exceed the World Health Organization's recommended daily intake of <2 grams of dietary sodium (equivalent to 5 grams of salt, sodium chloride) per day for adults [5]. Processed foods, restaurant foods, and discretionary salt and sauce intake contribute considerably [6].

Furthermore, limiting sodium intake in those with CKD is paramount as the kidney has decreased capacity to excrete sodium and water. Current Kidney Disease Improving Global Outcomes (KDIGO) guidelines also recommend limiting daily sodium intake to <2 grams for individuals with CKD [7]. As the ability to excrete sodium decreases with progressive kidney decline [8], the negative impact of sodium on health outcomes is accentuated, with higher sodium intake linked to CKD progression [9], worsening hypertension, cardiovascular mortality and morbidity [10], increasing risk of left ventricular hypertrophy [11], and vascular stiffness [12]. Sodium has also been reported to influence uraemia-related risk factors via oxidative stress pathways leading to inflammation, endothelial cell injury, and proteinuria [13, 14]. As CKD prevalence continues to rise globally [15], improving the management of sodium balance is important to address the increased cardiac mortality and morbidity. This begins with more accurate measurement of sodium intake, excretion, and removal.

### 2.2. Sodium Physiology and Pathophysiology in CKD

To better understand the current measurements of sodium, their strengths and limitations, and the utility in the population with CKD, one first requires a model of sodium homeostasis and physiology and the impact of CKD on this. Renal regulation of sodium, through a complex interaction of mediators involving the sympathetic nervous system, renin-angiotensin-aldosterone system, and natriuretic peptides, is required to maintain sodium homeostasis in healthy physiological states [16]. This tightly controlled system is disrupted with decreasing glomerular filtration rate and progression of CKD that increasingly compromises the kidneys' concentrating capacity [17]. With an overall decrease in functioning nephron mass, there is a compensatory increase in the fractional sodium load excretion per nephron unit with the consequent insult of increased osmotic diuresis [17]. Volume-dependent and independent factors influencing atrial natriuretic peptide increase the renal blood flow and decrease sodium reabsorption in the distal tubules [18, 19]. With the progression of renal decline, these adaptive mechanisms are overridden leading to a net salt excess, hypertension, and clinically evident fluid overload [17]. However, some observational studies have demonstrated that high sodium intake or positive sodium balance does not correlate with a concomitant increase in extracellular volume, total body weight, or rise in serum sodium in healthy individuals [20, 21]. This has been explained by a greater appreciation of extrarenal sodium handling in tissues (skin and muscle) over the last two decades [1]. This further complicates the understanding of human sodium physiology in those with CKD.

How sodium is stored and regulated in tissues is not entirely clear and requires further investigation. In some studies of rodent and human tissues, sodium has been demonstrated to be bound to the glycosaminoglycan- (GAG-) rich tissue interstitium in a water-independent manner [22, 23]. The electrostatic interaction between negatively charged GAG and sodium inactivates the osmotic effects of excess sodium and prevents the extracellular fluid expansion [23]. Dermal sodium is correlated with increasing GAG content in the skin [24]. In a hypertonic microenvironment, skin phagocytes activate tonicity enhancer binding proteins and vascular endothelial growth factors (VEGF-C) mediating lymphangiogenesis. This modulates cutaneous lymph capillary density and induces local tissue electrolyte clearance to regulate skin sodium levels [25]. This water-free storage of tissue sodium has been consolidated by ^1^H and ^23^Na MRI images that can colocate sodium and water in Kopp et al.'s work, which demonstrated an age-dependent increase in male muscle content without an associated increase in serum sodium or water content and volume expansion [24]. Additionally, the role of epithelial sodium channel (ENaC) in endothelial, vascular smooth muscle, and immune cells, like monocytes, that mobilise in and out of tissues has also been an area of interest involved in extrarenal blood pressure regulation [26]. Hydration levels through mediation by ENaC can influence dermal fibroblasts and skin inflammatory responses [27]. Skin provides an environment for cellular crosstalk, similar to the kidney. Endothelial cells can act as vascular salt sensors and modulate the mechanical stiffness of the cell plasma membrane by also activating ENaC and nitric oxide release in response to sodium concentrations in plasma [28]. However, others have posited that the increased tissue sodium signal is related to a shift of sodium intracellularly in exchange for potassium [29] or a systemic change in the extracellular to intracellular compartments with isotonic sodium accumulation in the extracellular space with concomitant water [30]. Therefore, more studies need to be conducted into the physiology of tissue sodium to better understand how this compartment relates to the totality of human sodium balance.

### 2.3. Current Urinary Measurement Methods of Sodium Balance: Strengths, Limitations, and Challenges in CKD

Current methods of measuring sodium intake rely on estimating sodium from dietary records or by excretion either with 24-hour urine collections or predictive equations from spot urinary sodium. Comprehensively collected 24-hour urine sodium (24 hUNa) is considered the gold standard for measuring dietary sodium intake [31], with various studies linking 24 hUNa to adverse outcomes in both the general population and those with CKD [32, 33]. However, 24-hour urine collections are cumbersome for patients and prone to undercollection [34]. Therefore, use of predictive equations to estimate 24 hUNa using spot urine tests have been developed as a more practical and repeatable means of measuring urinary excretion. Predictive equations to estimate 24 hUNa using spot urine tests in the non-CKD population are summarised in Table 1. Dysregulated sodium physiology in CKD complicates the accuracy of urine sodium measurement. For example, total sodium urinary output is decreased in stage 5 CKD compared with a less advanced CKD [35].  Table 2 outlines studies comparing two methods of urinary sodium measurement in patients with CKD (24 hUNa vs. predictive equations to estimate 24 hUNa using spot urine tests) published in indexed journals over the last twenty years [36–39].

#### 2.3.1. Estimates of 24-Hour Urinary Sodium Collections from General Populations

Three studies included in Table 2 evaluated the Tanaka and/or Kawasaki predictive equations, derived from non-CKD cohorts, in CKD populations of Japan [40–42], where dietary sodium intake is relatively higher [43]. The mean estimated glomerular filtration rate (eGFR) in these four studies ranged widely from 31 to 53 mL/min/1.73 m^2^. There was generally an equal ratio of males to females except for the study by Imai et al., which involved 72.1% males [41]. The Tanaka equation was more accurate in Japanese patients with CKD compared with the Kawasaki equation in the study by Imai et al. [41]. However, this study collected first morning urine samples, which may be more practical, whereas the Kawasaki equation utilises the second morning urine sample. Similarly, Ogura et al. reported that estimated sodium excretion using Tanaka's method (developed from INTERSALT Japanese data) significantly correlated with measured sodium excretion in another cohort of Japanese patients with stages 1 to 5 CKD [42]. Estimated sodium excretion correlated better in those with eGFR <30 mL/min and when the sodium excretion cut-off point was >170 mEq/day. This suggests Tanaka's estimation in a Japanese CKD population is more accurate at the lower range of kidney function and higher sodium intake. In contrast, Amano et al. reported considerable differences between 24 hUNa compared with sodium excretion estimated from untimed spot urine samples using the Tanaka formula. This study was also conducted in a CKD Japanese population but involved a higher mean eGFR of 42.8 ± 23.9 mL/min/1.73 m^2^ [40]. Dougher et al. similarly showed that the Nerbass, INTERSALT, and Tanaka equations underestimated sodium excretion when dietary intake was high [44]. These four studies emphasise that predictive equations derived from healthy cohorts are not validated in heterogeneous CKD populations and the accuracy of these predictive equations may be affected by level of kidney function.

Furthermore, there are other factors that influence measured sodium in spot urine samples. Accuracy of urine sodium estimation seemed dependent on the collection time of the spot urine sample in those with CKD. The normal circadian pattern of electrolyte and water excretion is disrupted in patients with CKD [45]. Nighttime urinary sodium excretion can be influenced by daytime exercise [46] and dietary sodium intake [47]. Elevated nocturnal blood pressure leads to higher nighttime urinary sodium excretion [48], whereby urinary sodium excretion can be up to 30% to 40% higher in those with eGFR <60 mL/min/1.73 m^2^ [49]. Timing of spot urine samples was inconsistent with the prescribed timing of spot urine samples in the original predictive equations (shown in Table 1) in the reviewed studies in Table 2. For example, the Kawasaki equation, designed for use with a second morning void, may have been affected by the use of an untimed spot urine sample in the study by Dougher et al. [44]. Therefore, utilising an inappropriately timed urine sample will distort the accuracy of spot urine sodium predictive equations to estimate 24 hUNa. It is also challenging to apply predictive equations using spot urine sodium samples to estimate 24 hUNa in populations that are geographically different to the sample populations from which they were derived. The Nerbass et al. [50] (developed from a stage 3 CKD population from the United Kingdom (UK)), INTERSALT [39] (developed from a healthy North American and European population), and Tanaka et al. [37] and Kawasaki et al. equations [38] (both developed from a healthy Japanese population) had poor precision and accuracy in estimating sodium excretion from untimed urine samples in patients with stages 3 and 4 CKD from the United States (US) [44]. This may be explained by differences in diet and body weight [51]. Alternatively, the use of a standardised spot urine potassium in the INTERSALT equation of this study instead of a randomly collected spot urine potassium may distort the predictive value. Decreasing kidney function, increasing age, and dietary restrictions would impact the creatinine and potassium values applied in the INTERSALT equation [52].

#### 2.3.2. Estimates of 24-Hour Urinary Sodium Collections from CKD Populations

Estimations of 24 hUNa from spot urine sodium levels specifically developed from CKD populations may more accurately reflect the physiology of kidney dysfunction compared with those developed in non-CKD populations. In patients with CKD, it is difficult to assess the degree of accuracy to which urinary sodium excretion approximates dietary sodium intake due to the disrupted handling of sodium by the kidney. We identified five 24 hUNa predictive formulae developed in CKD populations in Table 2 [50, 53–56]. However, kidney dysfunction was not severe. Hu et al. developed the CKDSALT formula with validation in patients with stages 1 to 4 CKD [53]. The CKDSALT equation showed lowest bias with limits of agreement compared with the Kawasaki, INTERSALT, and Tanaka formulae. However, this method requires 24-hour urine volume as an equation variable, which suggests that the decreased urine volume in patients with CKD has an important effect on estimating urinary sodium in those with CKD. This study highlights the potential to strike a balance between practicality and accuracy with predictive equations by simply recording urine volume without the cumbersome need to collect the urine. Hu et al. also found utilising first morning urine in the Kawasaki equation led to overestimation in patients with CKD. Thus, the predictive accuracy of the Kawasaki equation may be affected by nocturnal natriuresis in CKD. Kang et al. showed that spot urinary sodium levels collected in the evening were 28% higher than in the morning, and the mean of three spot urinary sodium samples was better correlated with 24 hUNa measurements compared with individual spot urinary sodium results in CKD patients [57]. Kim et al. developed the ESPECIAL formula in a young Korean cohort with stages 1 to 3 CKD [54]. This formula was more reliable in predicting 24 hUNa using fasting morning urine samples compared with Kawasaki, INTERSALT, Tanaka, and Nerbass formulae in this cohort of mild-moderate kidney dysfunction. As such, this ESPECIAL formula may not be as accurate in more severe stages of CKD. Nerbass et al. developed a new formula in stage 3 CKD patients from the UK using an early morning urine. In comparison, Tanaka's formula was only weakly correlated in the same study population [50]. Nerbass et al. also developed a new formula to estimate 24 hUNa in those with a high sodium intake (>3.6 g/day) from a second void spot urine sample of Brazilian patients with CKD [55]. These two studies by Nerbass et al. demonstrated that estimates are sensitive in high sodium consumption in the CKD population, which may be clinically useful in assessing high sodium intake in large cohort or observational studies. In contrast, Subramanian et al. developed a model for predicting 24 hUNa <100 mmol in a multiethnic Asian population with and without CKD [56]. It is inherently difficult to take into account variable ages, diuretic use, urine volume and creatinine excretion, stages of CKD, and individualised diet and fluid restrictions when developing a predictive equation to estimate 24 hUNa from spot urine sodium samples in CKD. Therefore, it is difficult to generalise these equations to a global heterogeneous CKD population with variable medications.

### 2.4. Nonurinary Measurements of Sodium Balance: Intake and Excretion/Removal

As can be appreciated, there is a need for innovative and practical methods of sodium measurement or monitoring. Other measurement methods to capture sodium removal, concentration, and tissue storage not using conventional urinary sodium measurements are outlined in Table 3. Urine chloride strips to self-monitor sodium intake were found to be significantly interrelated with 24 hUNa [58]. Participants in this study had predominantly stage 3 CKD, with a mean eGFR of 40.4 mL/min/1.73 m^2^. However, other sources of dietary chloride, besides sodium chloride, can cloud the results, and the chloride strips were only sensitive at identifying patients with 24 hUNa >100 mmol/day. Although patients found this self-monitoring of high sodium intake technique useful, this method requires patients' motivation to collect 24-hour urine volumes for self-testing, which can be cumbersome.

Patients with kidney failure on dialysis rely on a dialyser and dialysate sodium gradients (in haemodialysis) or peritoneal membrane and peritoneal fluid composition (in peritoneal dialysis) to remove sodium. A small cohort of patients on haemodialysis was assessed for sodium removal by the dialyser compared with sodium retrieval in ultrafiltrate and dialysate [59]. There was no relationship between measured sodium removal from the vascular space and tissue sodium removal and no relationship between dialysate/plasma sodium concentration gradients and tissue sodium removal. This suggests that the secondary clearance of tissue sodium is quantitatively less predictable despite successful intravascular sodium removal. Hence, we need to quantify the sodium pool in tissue *in vivo*.

Dietary records are another way of measuring sodium intake. However, only three published studies in the CKD population have reported sodium dietary intake in the last two decades [57, 60, 61]. Hallvass et al. compared 24 hUNa with total sodium consumption in a three-day food record (two consecutive days and one day of the weekend) in CKD patients with a mean eGFR of 38.9 ± 15.4 mL/min/1.73 m^2^ [61]. These records were checked by a dietician with software calculation. Calculations also included discretionary sodium, which can be hard to measure. They found a comparable mean sodium consumption (4.49 ± 2.19 g/24 h) in CKD to measured 24 hUNa (4.14 ± 1.71 g/24 h). Similarly, Kim et al. found that a one-day diet diary (assessed by a dietician that included discretionary sodium) significantly correlated with 24-hour sodium removal measurements (the sum of 24 hUNa and peritoneal sodium removal) in a younger cohort of patients on peritoneal dialysis (mean age <60 years) [60]. This study was opportunistic and minimally burdensome to patients who routinely collected urine volumes for dialysis adequacy assessment. Notably, dietetic resources may be more readily available in clinical studies. Kang et al. also found that mean spot urinary sodium tended to be better correlated with 24 hUNa compared with sodium intake recorded on dietary recall [57]. Sodium intake estimates based on food diaries, food-frequency questionnaires, and 24-hour or multiday recall are relatively easy to conduct. However, they tend to underestimate sodium intake compared with estimates obtained from duplicate diets and 24-hour urine collections [62]. Sodium intake estimates are inherently susceptible to recall and reporting bias and are interviewer-dependent [63, 64].

### 2.5. Limitations of Current Sodium Balance Measurements

The heterogeneity in the studies in Tables 2 and 3 highlights the limitations of current measurements including inaccuracies and challenges in clinical practice. Although it is of low cost, 24 hUNa comes with inherent challenges of high participant burden and susceptibility to inaccurate collection in clinical practice. Completeness of 24 hUNa collection was unclear in some of the studies included in this review. Varied provision of verbal and/or written collection instructions potentially affects urine collection completeness [65]. Up to half of 24-hour urine collections are undercollected [34], and only three studies assessed urine collection adequacy with creatinine clearance criteria [42, 50, 57]. Attempts to capture interday variation with repeat 24-hour measurements in patients with stages 3 and 4 CKD at regular intervals have been compared with single 24 hUNa measurements with some success [44]. However, 24-hour urinary creatinine excretion also demonstrates intradaily variation affected by protein intake, exercise, and muscle mass [66, 67]. Measurement of urinary creatinine excretion in CKD is further complicated by considerable muscle mass wasting and low glomerular filtration [68]. With these limitations, it is difficult to define a gold standard to measure urinary sodium in CKD though there has been a reliance on 24-hour urine collections as this remains a practical option in clinical practice. Spot urine tests are less cumbersome and more practical to collect. However, accuracy varies when predictive equations are applied to CKD cohorts, depending on origin of predictive model. Shorter durations (less than 24 hours) of urine collection, which increase collection completeness and decrease participant burden, may strike a balance. Equations estimating 24 hUNa derived from 12-hour urine collections performed better than spot urines in patients with CKD [69]. A timed period of collection may negate intraday spot urine sodium excretion variation. Additionally, the aforementioned measurement methods also do not account for extrarenal losses or tissue sodium content, neglecting a clinically significant part of body sodium composition.

### 2.6. Evaluating Tissue Sodium Content

Visualisation of sodium tissue content by sodium-23 magnetic resonance imaging (^23^Na MRI) has shown the potential of supplement measurements of sodium dietary intake and urinary sodium excretion to better reflect whole body sodium levels [1]. ^23^Na MRI has been used to characterise sodium content in different body areas to understand human pathology. Currently, ^23^Na MRI characterises human tissue sodium concentrations typically using a dedicated knee coil to acquire quantitative sodium images of calf tissue (skin and muscle) [70].

Studies visualising the sodium tissue signal with ^23^Na MRI have demonstrated that tissue sodium is correlated with age [24, 59, 71–73] and male gender [24, 71–74] in healthy controls as well as those with CKD. In CKD, tissue sodium concentrations increase progressively across the spectrum [74]. Patients with kidney failure on haemodialysis and peritoneal dialysis have significantly higher sodium concentrations in the skin, soleus, and tibia compared with controls [74]. Sodium quantification by ^23^Na MRI demonstrated that patients with refractory hypertension had significantly increased sodium content compared with normal controls, when controlled for age [24]. In patients with primary hyperaldosteronism, surgical or medical treatment with spironolactone for hypertension decreased muscle sodium content by 30% (26.1 ± 3.1 vs. 18.3 ± 2.7 mmol/L) with no effect on body weight, demonstrating that tissue sodium content is mobilizable [72].

As tissue sodium storage has been demonstrated to be exchangeable, it could be said that intraindividual tissue sodium could change across time, but further studies are required to explore this. There has yet to be studies of serial ^23^Na MRI in individuals without interventions to ascertain an understanding of the natural physiological variations in tissue sodium content. Other studies have explored the effect of haemodialysis [54, 59] and altering haemodialysis dialysate sodium concentration [75] on tissue sodium content, but other confounding factors that can influence tissue sodium content need exploration across the spectrum of CKD and dialysis modalities. Such studies could have important implications for novel therapeutic targets to reduce total body sodium content and improve clinical outcomes.

Clinical associations of tissue sodium content are in the early stages of being elucidated in small studies. Tissue sodium is associated with inflammation, immune dysregulation, and hypertension, independent of circulating osmotically active sodium [24]. Skin sodium concentration correlates with left ventricular mass, independent of blood pressure and overhydration [71]. Studies have also yet to delineate the implications of tissue sodium on clinical outcomes, like cardiovascular mortality independent of osmotically active sodium in the extracellular space. Overall, these human studies evaluating tissue sodium concentration using ^23^Na MRI do not have any controlled dietary interventions and have not correlated findings with sodium dietary intake measurements or urinary sodium excretion (routinely available methods in current clinical practice).

A detailed clinical cost analysis and efficacy study would be required if indeed MRI will provide additional data in the overall assessment and management of a patient in the clinical setting. Although requiring technical expertise, time, and higher costs than current sodium intake measurements, ^23^Na MRI is an emerging clinical tool that has the potential to supplement current sodium measurements in clinical studies to better understand body sodium balance. Given salt's dramatic negative consequences on health outcomes, it would be vital to explore the clinical associations of tissue sodium and novel therapeutic targets.

## 3. Conclusion

Current measurements of sodium urinary excretion and dietary intake do not reflect the total body sodium balance. Twenty-four-hour urine collections are considered more accurate than spot urine sodium measurements but are also onerous for patients. Equations to estimate 24 hUNa from spot urine sodium samples developed from healthy populations are not reliable in heterogeneous CKD patients. Predictive equations developed specifically in CKD cohorts require further validation in different CKD stages and ethnic and geographical groups. These predictive formulae are also not applicable for individual sodium assessment as they were developed in larger scale cohort and epidemiological studies. Dietary records are inherently prone to recall bias and not reflective of interday variation.

Comprehensive and physiologically accurate measurements of sodium balance need to include tissue sodium levels. The advent of ^23^Na MRI adds further weight to extrarenal sodium handling. However, more studies are needed to compare tissue sodium concentrations with current measurements of sodium intake and sodium urinary excretion. Future larger prospective studies can evaluate the sodium reduction strategies and associated clinical outcomes using ^23^Na MRI that measure changes in tissue sodium content. We need to better understand and ameliorate the negative clinical impacts of tissue sodium, especially in patients with CKD, as there have been little interventional advances in this space in recent times. Utilising novel imaging technology may help pave the way.

## Figures and Tables

**Table 1:** Predictive equations developed from non-CKD populations to estimate 24-hour urinary sodium excretion from spot urine sodium samples.

Equation	Country	Sample	Urine sample collection	Other equation variables
Kamata and Tochikubo [36]	Japan	126 healthy men (mean age years ± standard deviation (38.0 ± 20.3]); 224 women (50.0 ± 16.0)	Overnight urine sample collected by semiautomatic proportional urine sampling device	Overnight urinary sodium/creatinine excretion ratio, age, sex, predicted 24-hour urinary creatinine excretion derived from body weight, height, and mass determined by bioelectrical impedance
Tanaka et al. [37]	Japan	295 healthy men (mean age years ± standard deviation (40.0 ± 11.1)); 296 women (39.0 ± 11.2)	One “casual” untimed spot urine	Predicted 24-hour urinary creatinine from age, weight, and height
		Developed from three Japanese populations from INTERSALT study		
		Examined accuracy of equation in 513 external manual workers		
Kawasaki et al. [38]	Japan	78 healthy men (mean age years ± SD (34.9 ± 1.1)); 81 healthy women (33.1 ± 1.3)	Second morning urine sample collected within four hours after the first void upon awakening but before breakfast, collected in a sitting/standing position	Predicted 24-hour urinary creatinine excretion based on age, body weight, height, and sex
INTERSALT, Brown et al. [39]	North America and Europe	5,693 participants with ages ranging from 20 to 59 years: randomly assigned to test (*n* = 2948) or validation (*n* *=* 2745) datasets	One “casual” untimed spot urine	Age, weight, height, and sex

**Table 2:** Studies comparing two urinary sodium measurement methods in patients with CKD (published from 2000 to 2021).

Year	Author	Country	Sample	Comparison	Findings
*Equations developed from non-CKD populations to estimate 24-hour urinary sodium excretion from spot urine sodium samples in CKD populations*					
2011	Imai et al. [41]	Japan	136 patients with CKD	24 hUNa vs. first morning SU (estimation using Kawasaki's and Tanaka's equation)	Sodium excretion estimated by Tanaka's equation can be used accurately in clinical practice. Nocturia, diuretics, ACEi, or ARB did not affect accuracy
2012	Ogura et al. [42]	Japan	334 urine samples from 96 patients with CKD stages 1–5	24 hUNa vs. SU on the same day that patients brought their 24-hour urine samples to the hospital (estimation using Tanaka's equation)	Estimated sodium excretion from spot urine samples significantly correlated with 24 hUNa using Tanaka's method
2016	Dougher et al. [44]	United States of America	129 patients with stages 3-4 CKD	Averaged multiple 24 hUNa collected over nine months vs. SU samples	All four estimation equations (Nerbass, INTERSALT, Tanaka, and Kawasaki) had poor precision and accuracy
2018	Amano et al. [40]	Japan	162 CKD: 72 patients, eGFR ≥60; 59 patients, eGFR 31–59; 31 patients, eGFR ≤30	24 hUNa vs. SU (estimation using Tanaka's equation)	Considerable difference between sodium excretion by 24 HU (2744 mg/day) and estimating SU (3315 mg/day)
*24-hour urine sodium excretion estimates from spot urine sodium samples in a CKD population*					
2012	Kang et al. [57]	Korea	305 patients with CKD: 48, stage 1; 67, stage 2; 79, stage 3; 58, stage 4; 53, stage 5	Mean of three SU (obtained on separate days one in the evening; one first urine in the morning; and one in the daytime) vs. 24 hUNa vs .sodium intake by dietary recall during the 24 hUNa collection period	Mean SU sodium can be used to monitor sodium intake in patients with CKD
					Mean SU sodium tended to be better correlated with 24 hUNa compared with sodium intake recorded on dietary recall

2013	Subramanian et al. [56]	Singapore	230 patients with CKD; 103 patients without CKD	24 hUNa vs. SU	Developed formula for predicting 24 hUNa <100 mmol/day
2014	Nerbass et al. [50]	United Kingdom	70 patients with CKD stage 3	24 hUNa vs. early morning SU on the day after completing the 24 HU	Developed formula that can be used in identifying high sodium intake in people with CKD
			74 additional samples from 50 patients with CKD stage 3 used to validate the formula		Significant correlations observed between 24 hUNa and early morning urine
2017	Nerbass et al. [55]	Brazil	51 patients with CKD stages 2–5	24 hUNa	Developed formula to identify sodium consumption >3.6 g/day (with high sensitivity)
				**vs.** SU (second urine of the day)	
2018	Kim et al. [54]	Korea	233 patients with CKD stages 1–3	24 hUNa vs. fasting morning SU on the day of the visit at 0, 8, and 16 weeks (obtained between 8 and 11 AM)	Developed ESPECIAL equation for estimating 24 hUNa excretion in CKD patients
					ESPECIAL equation more reliable in predicting 24 hUNa in CKD patients using fasting morning SU compared with Kawasaki, INTERSALT, Tanaka, and Nerbass formulas
2019	De Vico Ribeiro et al. [69]	Brazil	76 CKD patients	24 hUNa vs. sodium excretion in 12-hour daytime and 12-hour nighttime collections; SU 1 (first urine of the day) and SU 2 (second urine of the day)	Equations developed from 12-hour urine collections performed better than spot urine when compared with gold-standard 24 hUNa
			51 additional CKD patients used to validate the equation developed from SU 2		Equation from SU 2 showed good sensitivity to identify excessive sodium intake
2020	Hu et al. [53]	China	5235 patients with CKD stages 1–4 (2460 derivation cohort; 2775 validation cohort)	24 hUNa vs. SU collected next morning after 24-hour urine collection	Developed CKDSALT equation for estimating 24 hUNa excretion in CKD patients
					CKDSALT equation had the best performance in any subgroup analysis compared with Kawasaki, INTERSALT, and Tanaka

24 hUNa: 24-hour urinary sodium excretion; 24 HU: 24-hour urine; ACEi: angiotensin-converting enzyme inhibitors; ARB: angiotensin II receptor blockers; SU: spot urine; HD: haemodialysis; PD: peritoneal dialysis; Na^+^: sodium.

**Table 3:** Studies exploring nonurinary sodium measurement methods in patients with CKD (published from 2000 to 2021).

Year	Author	Country	Sample	Comparison	Findings
*Dietary records estimating sodium intake in CKD populations*					
2015	Hallvass et al. [61]	Brazil	60 patients with CKD not on renal replacement therapy	24 hUNa vs. a three-day food record (two consecutive days and one day of the weekend)	Comparable mean sodium consumption (4.49 ± 2.19 g/24 h) in CKD to measured 24 hUNa (4.14 ± 1.71 g/24 h)
2017	Kim et al. [60]	Korea	86 PD patients, divided into 44 RRF (+) and 39 RRF (−) patient	One-day diet record vs. total sodium removal (sum of peritoneal sodium added to the amount of sodium in the 24 HU)	Significant positive correlations between sodium intake and total sodium removal in both groups RRF (+) and RRF (−)
Also see Kang et al. [57] Table 2					

*Nonurinary measurements of sodium excretion/removal in CKD populations*					
2015	Dahlmann et al. [59]	Germany	24 HD patients and 27 age-matched healthy controls	Calculated Na^+^ removal by the dialyser vs. Na^+^ retrieval in ultrafiltrate and dialysate	Calculated sodium removal by the dialyser highly correlated with sodium retrieval in ultrafiltrate and dialysate
			Subgroup of 20 HD patients before and shortly after HD with a batch dialysis system		No linear relationship between measured sodium removal from vascular space or dialysate/plasma [Na^+^] gradients and tissue Na^+^ removal
2019	Panuccio et al. [58]	Italy	72 CKD patients: 4, stage 1; 12, stage 2; 35, stage 3; 14, stage 4; 7, stage 5	24 hUNa vs. urine chloride strips for self-monitoring sodium intake from a 24 HU collection	Self-measurement of urine chloride with reactive strips to estimate 24 hUNa had reasonable sensitivity (75.5%) and specificity (82.6%) for excessive sodium chloride intake (>100 mmol/day)

24 hUNa: 24-hour urinary sodium excretion; 24 HU: 24-hour urine; SU: spot urine; HD: haemodialysis; PD: peritoneal dialysis; Na^+^: sodium; RRF: residual renal function (defined as urine output >100 ml/day).
